# Associations Between Domains and Patterns of Sedentary Behavior with Sleep Quality and Duration in Pregnant Women

**DOI:** 10.3390/healthcare13030348

**Published:** 2025-02-06

**Authors:** Nada Khojah, Bethany Barone Gibbs, Saja Abdullah Alghamdi, Alawyah Alsalman, Om Kalthom Sowadi, Hadeel Saad, Ghareeb Omar Alshuwaier, Abdullah Bandar Alansare

**Affiliations:** 1 Department of Exercise Physiology, College of Sport Sciences and Physical Activity, King Saud University, King Khalid Rd, Riyadh 11451, Saudi Arabia; khojahnada@gmail.com (N.K.); sajaalghamdi19@gmail.com (S.A.A.); omkalthom.sowadi@hotmail.com (O.K.S.); hadeel.f.saad@gmail.com (H.S.); galshuwaier@ksu.edu.sa (G.O.A.); 2 Department of Epidemiology and Biostatistics, West Virginia University School of Public Health, Morgantown, WV 26506, USA; bethany.gibbs@hsc.wvu.edu; 3 Department of Physical Education, College of Sport Sciences and Physical Activity, King Saud University, King Khalid Rd, Riyadh 11451, Saudi Arabia; alwyah2020@gmail.com

**Keywords:** pregnancy, midwifery, women’s health, leisure, occupational, commuting, lifestyle, sitting

## Abstract

**Objectives:** To assess the associations of sedentary behavior (SB) with sleep quality and duration in pregnant women by trimesters and to consider SB domains and patterns. **Methods:** This investigation included 935 participants (age = 30.0 ± 5.6 years; first trimester = 24.1%, second trimester = 33.9%, third trimester = 42.0%). The total, domains (leisure, occupational, commuting), and patterns (weekdays, weekends) of SB, as well as sleep quality and duration, were self-reported. Adjusted logistic regression models examined the associations of different SBs with the risk of poor sleep quality, not adhering to sleep duration guidelines (7–9 h/day), or being a short- or long-sleeper. **Results:** The odds of poor sleep quality were significantly higher by 14.2% and 7.4% for each hour increase in total and leisure SB on weekends, respectively. The odds of not adhering to sleep duration guidelines were significantly higher by 5.5% and 11.4% for each hour increase in total and leisure SB during weekdays, respectively; paradoxically, the odds of not complying with the sleep duration guidelines were significantly lower, ranging between 6.6% and 34.4% for each hour increase in total, leisure, or commuting SB on weekends. Further, when analyzed separately, the likelihood of being a short- or long-sleeper was highly variable across SB domains, with associations being more apparent in the first and third trimesters. **Conclusions:** The relationship between SB and healthy sleep during pregnancy was complex. These variable findings underscore the importance of considering domains and patterns of SB beyond simple total durations in future research to inform interventions and guidelines to improve sleep health during pregnancy.

## 1. Introduction

Healthy sleep is now recognized as a vital behavioral component of cardiovascular health [1]. Sleep duration that is too short (<7 h per night) or too long (>9 h per night), as well as poor sleep quality, has been associated with many adverse health outcomes, including metabolic syndrome, diabetes, autonomic dysfunction, and cardiovascular disease (CVD) [2,3,4]. Poor sleep quality and insufficient sleep duration have also been associated with an increased risk of CVD-related pregnancy complications, perinatal and postpartum depression, and fetal health issues [5,6,7,8]. Noticeably, inadequate sleep is frequently observed among pregnant women. For instance, a recent meta-analysis that included 11,002 pregnant women found that 45.7% reported poor sleep quality [9]. Furthermore, 29% to 45.5% of pregnant women worldwide suffered from insufficient sleep duration (i.e., not acquiring 7–9 h) [10,11,12]. As a result, the effectiveness of various pharmacological (e.g., benzodiazepine receptor agonists) and non-pharmacological (e.g., exercise) interventions on sleep during pregnancy has been investigated and found to be effective [13,14]. However, pharmacological treatments are less desirable during pregnancy and pregnant women have many unique barriers to exercise [15,16]. Therefore, exploring alternative non-pharmacological approaches that may promote adequate and high sleep quality during pregnancy is warranted.

Sedentary behavior (SB) is any waking behavior with low energy expenditure that occurs while seated, lying, or reclining [17]. Pregnant women spend over 50% of their time engaged in SB [18], with nearly 20% of this time accumulating in prolonged sessions lasting 30 min or more [19]. Of note, SB has not only been recognized as a significant risk factor for CVD in general populations [20], but emerging research suggests that it may also be associated with pregnancy complications [18,21]. For example, higher SB has been associated with elevated prenatal blood pressure [22], gestational C-reactive protein [23], risk of fetal macrosomia [24], and adverse pregnancy outcomes (e.g., hypertensive disorders of pregnancy) [25]. The growing evidence linking SB with pregnancy-related outcomes has brought global attention to the importance of reducing excessive SB for healthy pregnancies [26,27].

Reducing time spent in SB may be a novel strategy for improving sleep health. A previous population-based cohort study observed that lower vs. higher SB was associated with enhanced sleep efficiency and lower odds of sleep apnea in the Swiss general population [28]. Recently, a large epidemiological study revealed that statistically exchanging one hour of SB with moderate-to-vigorous physical activity (MVPA) improved sleep quality (i.e., rest by sleep) in Japanese women [29]. However, whether time spent engaged in SB is related to sleep quality and duration in pregnant women has not been well studied. Given the limitation of current interventions to improve sleep during pregnancy [15,16], a comprehensive understanding of the relationships between SB and sleep quality and duration during pregnancy may facilitate the design of more optimal non-pharmacological interventions. Further, given that the associations of SB with sleep duration and quality in non-pregnant adults appear to vary based on the context or domain within which the SB is accumulated (e.g., leisure, occupational, commuting) and the pattern of SB (weekday vs. weekend), it is critical that such investigations in pregnancy women consider these nuances [30].

Therefore, the aims of this investigation were to (1) assess the associations of total, leisure, occupational, and commuting SB with sleep quality and duration in pregnant women by trimesters and (2) to examine the role of SB patterns (i.e., weekend vs. weekday). Further, an exploratory aim was to assess whether total and domain-specific SB was associated with the likelihood of being a short- or long-sleeper.

## 2. Materials and Methods

This secondary investigation utilized data from a recently published study [12] conducted according to the Declaration of Helsinki. The reporting in the current manuscript was completed by following Strengthening the Reporting of Observational Studies in Epidemiology (STROBE) guidelines. Details of participant recruitment are reported in detail elsewhere [12], but, briefly, cross-sectional data collection was completed between 3 July 2023 and 24 August 2023 in Saudi Arabia. Research team members visited private and public hospitals and clinics and were assigned to recruit and administer questionnaires to pregnant women of any gestational age in waiting areas at obstetrics and gynecology clinics. To be enrolled in the study, women had to be pregnant and permanently living in Saudi Arabia.

As the original study [12] intended to include a sample that represents women in Saudi Arabia, a proper sample size calculation was performed using the Raosoft calculator (a web-based calculator) with the following criteria: a population size of 15,429,586 (according to the 2022 World Bank Data), confidence level of 95%, margin of error of 5%, and power of 80%. The needed sample size was estimated to be 385. However, to improve the precision and generalizability of the results, a higher number of pregnant women were recruited.

Initially, 952 pregnant women were enrolled. As the original study aimed to assess the prevalence of adherence to sleep, SB, and physical activity (PA) recommendations, any participant who had unreasonable (i.e., >24 h/day) sleep (n = 2), total SB (n = 2), or PA (n = 11) were excluded. Moreover, pregnant women who reported unreasonable gestational ages (i.e., 63 weeks) (n = 1) or had missing body heights and/or weights (n = 1) were excluded. The exclusion of these participants (n = 17) was performed to reduce the effects of outliers or influential data points. Thus, the total number of participants included in the original study and the current secondary analysis was 935.

### 2.1. Measurements

#### 2.1.1. Maternal Demographic and Health-Related Variables

Demographic and health-related variables were self-reported (Table 1). Participants (n = 935) had an average age of 30.0 ± 5.6 years. Moreover, 24.1% (n = 225), 33.9% (n = 317), and 42.0% (n = 393) of these participants were in their first, second, or third trimesters, respectively. The majority of these pregnant women had at least a Bachelor’s degree (58.7%), at least one child (61.9%), were housewives (76.8%), and were largely healthy without a pre-existing chronic disease (91.2%). The average reported time spent in sleep was 7.26 ± 2.1 h/day, and participants reported an average of 6.8 ± 3.5 h/day of SB.

#### 2.1.2. Sleep Quality and Duration Assessment

Sleep quality and duration were estimated using the standardized Arabic version of the Pittsburgh Sleep Quality Index (PSQI) [31], a valid and reliable instrument for assessing sleep, including among pregnant women [32]. The PSQI consists of 9 items summed into 7 components (i.e., subjective sleep quality, sleep latency, sleep duration, sleep efficiency, sleep disturbance, use of sleep medication, and daytime dysfunction). Each component can have a score of 0 to 3 points. The scores of all components are aggregated to calculate the global index, which ranges from 0 to 21 points. Poor sleep quality was determined based on standard convention if the global index scored 5 points or higher [33]. Furthermore, sleep duration (hours/day) was estimated from a single self-report item on the PSQI. Meeting sleep duration guidelines was determined if the participants accumulated 7 to 9 h/day of sleep. Sleep duration was also used to categorize pregnant women as optimal (7 to 9 h/day), short (<7 h/day), or long (>9 h/day)-sleepers [34].

#### 2.1.3. Total and Domain-Specific SB Assessment

Total, leisure, occupational, and commuting SB (hours/day) during weekdays and weekends were estimated using the standardized Arabic version of the Sedentary Behavior Questionnaire (SBQ) [35]. The validity and reliability of the SBQ in pregnant women have also been established [36]. The SBQ queries seven types of leisure SB (i.e., sitting and reading a book or magazine, engaging in artwork or crafts, playing a musical instrument, sitting and listening to music, sitting and talking on the phone, playing computer or video games, and watching TV), one type of occupational SB (i.e., undertaking paperwork or computer work), and one type of commuting SB (i.e., sitting and driving a car, bus, or train) [37]. To calculate total SB during a weekday and weekend day, time spent engaged in all types of SB during a weekday and weekend day were aggregated separately. Then, total SB per day was computed as follows: total SB per day = ([total SB during a weekday × 5] + [total SB on a weekend day × 2])/7. Domain-specific SB was similarly calculated. Times spent in each domain (i.e., leisure, occupational, or commuting) of SB during a weekday and weekend day were summed separately [38]. The average domain-specific SB per day was calculated as follows: domain-specific SB per day = ([domain-specific SB during a weekday × 5] + [domain-specific SB on a weekend day × 2])/7.

#### 2.1.4. Moderate-to-Vigorous Physical Activity Assessment

To account for the influence of physical activity on the relationships between SB and sleep quality and duration in pregnant women, the standardized Arabic short version of the International Physical Activity Questionnaire (IPAQ) was used to estimate MVPA (min/day) [39]. The validity and reliability of the IPAQ in pregnant women also were previously established [40].

### 2.2. Statistical Analyses

Maternal demographic and health-related variables were reported as mean ± standard deviation or frequency and percentage, as appropriate. Binary logistic regression models examined whether higher total and domain-specific SBs were associated with higher odds of poor sleep quality and not adhering to the sleep duration guidelines. First, simple logistic regression models were fitted that associated one predictor at a time (e.g., total SB or leisure SB) with the binary outcome (i.e., poor sleep quality or not adhering to the sleep duration guidelines). Then, adjusted logistic regression models added covariate adjustments for demographic (i.e., age, having children, education, occupation) and health-related (i.e., smoking status and chronic disease status) variables, MVPA, and other SBs. These covariates were controlled based on previous research which identified them as important confounders for pregnant women [25,41,42]. Analyses were performed using JASP software (JASP 0.15 Version). Lastly, multinomial logistic regression models assessed whether higher total and domain-specific SBs were associated with higher odds of being a short-sleeper or long-sleeper as compared to an optimal-duration-sleeper. The later models were constructed using SPSS software (Version: 28.0.0.0.190). The significance level for all analyses was set at *p*-value < 0.05.

## 3. Results

Table 2 displays the associations of total, weekday, and weekend day SB, including domain-specific SB, with sleep quality. The odds of having poor sleep quality were significantly higher by 7.4% for each hour increase in leisure SB on weekends only (*p* < 0.05). When the trimesters were considered, a significant relationship was observed in pregnant women in their first trimester. The odds of having poor sleep quality were significantly higher by 14.2% for each hour increase in total SB on weekends only (*p* < 0.05). No other significant associations were detected between other SB and sleep quality (*p* > 0.05).

Table 3 shows the relationships between total, weekday, and weekend day SB, including domain-specific SB, with the odds of not meeting the sleep duration guidelines. The odds of not adhering to the sleep duration guidelines were significantly higher by 5.5% and 7.3% (*p* < 0.05 for both) for each hour increase in total and leisure SB during a weekday, respectively. In contrast, the odds of not adhering to the sleep duration guidelines were significantly lower by 8.0%, 6.6%, and 13.7% (*p* < 0.05 for all) for each hour increase in total, leisure, and commuting SB on a weekend day, respectively. When the trimesters were considered, further significant associations were detected in pregnant women in their first or third trimesters. The odds of not adhering to the sleep duration guidelines were significantly higher by 10.9% and 11.4% (*p* < 0.05 for both) for each hour increase in total and leisure SB during a weekday, respectively, only in the third trimesters. On the contrary, the odds of not adhering to the sleep duration guidelines were significantly lower by 34.4% for each hour increase in commuting SB per day only in pregnant women in their first trimesters (*p* < 0.05). Moreover, the odds of not adhering to the sleep duration guidelines were significantly lower by 13.2%, 10.9%, and 24.6% (*p* < 0.05 for all) for each hour increase in total, leisure, and commuting SB on a weekend day, respectively, only in the third trimesters. No other significant associations were detected between other SB and sleep duration (*p* > 0.05).

Table 4 reveals the associations of total and domain-specific SB with the odds of being a short-sleeper or long-sleeper versus an optimal-duration-sleeper. The odds of being a short-sleeper were significantly higher by 5.4% (*p* < 0.05) for each hour increase in total SB during a weekday. Conversely, the odds of being a short-sleeper were significantly lower by 4.3%, 22.4%, 11.3%, 8.1%, and 14.7% (*p* < 0.05 for all) for each hour increase in total and commuting SB per day and total, leisure, and commuting SB on a weekend day, respectively. Furthermore, the odds of being a long-sleeper were significantly higher by 5.5%, 12.1%, and 13.1% (*p* < 0.05 for all) for each hour increase in total SB and leisure per day and leisure SB during a weekday, respectively. In contrast, the odds of being a long-sleeper were significantly lower by 38.1% (*p* < 0.05) for each hour increase in occupational SB per day.

When the trimester was considered, further complex paradoxical relationships were detected only for pregnant women in their first or third trimesters. For pregnant women in their first trimesters, the odds of being a short-sleeper were significantly lower by 12.9%, 43.8%, and 16.8% (*p* < 0.05 for all) for each hour increase in total and commuting SB per day and total SB on a weekend day, respectively. On the other hand, the odds of being a long-sleeper were significantly higher by 23.2% and lower by 45.2% (*p* < 0.05 for both) for each hour increase in leisure SB per day and commuting SB during a weekday, respectively. For pregnant women in their third trimesters, the odds of being a short-sleeper were significantly higher by 9.4% and lower by 13.1%, and 28.7% (*p* < 0.05 for all) for each hour increase in total SB during a weekday and total and commuting SB on a weekend day, respectively. Moreover, the odds of being a long-sleeper were significantly higher by 11.9% and 20.6% (*p* < 0.05 for both) for each hour increase in leisure SB per day and during a weekday, respectively.

## 4. Discussion

This unique study is the first to our knowledge to evaluate associations between domains and patterns of SB with sleep quality and duration in pregnant women. The results were summarized in Table 5. Higher total or leisure SB, particularly on weekends, were associated with a higher risk of poor sleep quality (i.e., the odds were higher by 7.4% to 14.2%). Yet, while greater total or leisure SB during weekdays were related to a greater risk of not adhering to the sleep duration guidelines (i.e., the odds were higher by 5.5% to 11.4%), higher total, leisure, or commuting SB on weekends were paradoxically associated with better adherence to sleep duration guidelines (i.e., the odds of non-adherence were lower by 6.6% to 34.4%). More variable associations were detected for the likelihood of being a short- or long-sleeping pregnant woman across SB domains and patterns, though more associations were apparent in the first and third trimesters. These findings suggest small and divergent relationships between some types of SB and sleep quality and duration. However, the inconsistent relationships make strong conclusions or direct recommendations for intervention strategies more difficult.

### 4.1. Strengths

This study has key strengths to be highlighted. First, the sample was large and the recruitment approach was comprehensive, resulting in good diversity with respect to geography, culture, age, socioeconomic factors, and representation across trimesters. These aspects strengthen the generalizability of our results. In addition, the SB (i.e., SBQ) and sleep (i.e., PSQI) measurement tools were multiple-item instruments that captured a wide spectrum of SB domains and patterns and various dimensions of sleep health. Together, these questionnaires allowed for a thorough investigation of the relationships between SB and sleep duration and quality in pregnant women.

### 4.2. Limitations

Caution should be taken when interpreting our findings because the pregnant women in our sample tended to be healthy, highly educated housewives. Given that pregnant women with other characteristics, such as pre-existing health conditions or lower education, have been documented to have poorer sleep health [41,42], the associations of SB and sleep quality and duration in such populations may differ from those observed in the current study. For example, highly educated pregnant women, especially those with a higher economic status, tend to have higher health literacy [43]. Improved health literacy may enhance awareness of the importance of reducing SB and attaining adequate sleep. Consequently, the inter-related associations between SB and sleep can be influenced. In addition, the distribution of these pregnant women across the three trimesters is not perfectly balanced, which may have introduced bias in the findings in regard to the trimesters with the highest sample sizes (i.e., third and second trimesters, respectively) [44]. Although the SBQ and PSQI uniquely assessed a wide range of SB and sleep variables, they were self-report instruments. Such tools are prone to inaccuracies in recall or reporting [36,45,46]. Future studies with device-based assessments of SB and sleep variables are needed to confirm the findings herein. Finally, the cross-sectional nature of this investigation precludes the establishment of temporal relationships. Though our analyses hypothesized that SB would be related to sleep quality and duration, it is also possible that poor sleep could influence SB. Future studies with longitudinal measures are needed to clarify these temporal relationships.

### 4.3. Associations of SB with Sleep Quality and Duration

A previous investigation found that higher leisure SB was related to poor sleep quality in non-pregnant adults [30]. Herein, the current study added novel findings and revealed that pregnant women who accumulate higher total (i.e., during the first trimester) or leisure SB on weekends have slightly higher odds of poor sleep quality. These associations might be explained by the increased exposure to factors associated with nocturnal SB on weekends. For instance, nocturnal TV viewing, a common SB during pregnancy [47], can interrupt the secretion of melatonin hormone, disturb the sleep–wake cycle [48], and delay bedtimes [49]. It is worth mentioning that the leisure domain of SB, which includes a large proportion of TV time, makes up the largest proportion of total SB on weekends [50,51]. These findings suggest that reducing total and leisure SB on weekends, potentially via minimizing TV viewing, may promote healthy sleep quality during pregnancy.

Novel, variable associations between domains and patterns of SB with sleep duration were observed, especially during early and late pregnancy. Mostly small associations were observed between higher total or leisure SB on weekdays and higher odds of not complying with sleep duration guidelines. On the other hand, higher total, leisure, or commuting SB per day or on weekends were weakly associated with better sleep duration (i.e., lower odds of not adhering to sleep duration recommendations). Given the paradoxical relationships of the domains and patterns of SB with sleep duration and sleep quality, it is difficult to identify which types of SB would be the best target in regard to improving sleep during pregnancy. Still, domain and pattern-specific associations of SB with health outcomes in general populations [52,53] and the variable findings in this report underscore the importance of considering types of SB beyond simple total durations in future research.

Lastly, the current study provides deeper insights into the relationships between domains and patterns of SB with less optimal sleep durations (short and long) during pregnancy. While total SB during weekdays was associated with a higher likelihood of being a short-sleeping pregnant woman, total and leisure SB per day during weekdays was associated with greater odds of being a long-sleeping pregnant woman. In contrast, higher total, leisure, or commuting SB per day or on weekends was associated with lower odds of being a short-sleeping pregnant woman, while higher occupational or commuting SB per day was associated with a lower likelihood of being a long-sleeping pregnant woman. All in all, this evidence further supports complex associations between SB and sleep duration classifications in pregnant women.

### 4.4. Clinical Implications

This study suggests that the type and patterns of SB are related to sleep quality and duration during pregnancy yet in variable ways. These initial findings indicate that modifying SB during pregnancy could potentially improve sleep quality and duration. These findings encourage medical professionals to monitor the domains and patterns of SB during pregnancy and advise pregnant women to minimize SB for better sleep health. As most current practices, interventions, and policies solely focus on SB or sleep for a healthier pregnancy, this study offers an opportunity to reconsider the simultaneous integration of SB and sleep modifications into practices to promote healthy pregnancies. Nonetheless, a clear targeted type and pattern of SB to reduce that could globally improve sleep quality and duration was not apparent. Rigorous longitudinal investigations with device-based measures of SB and sleep and, eventually, randomized controlled trials testing SB-reduction interventions are needed to inform clinical guidelines.

## 5. Conclusions

In summary, this investigation reports the initial relationships between domains and patterns of SB with sleep quality and duration in pregnant women. As several paradoxical associations were detected between different domains and patterns of SB with sleep quality and duration, these results suggest that SB may have small associations with healthy sleep during pregnancy but in complex ways. These variable findings highlight the significance of considering types and patterns of SB beyond simple total durations during pregnancy. Given the current study’s limitations, such as the cross-sectional nature and self-report instruments, definitive and clear recommendations or strategies for improving sleep during pregnancy with SB reduction are challenging. Further rigorous investigations that are longitudinal and use device-based measures of SB and sleep may identify stronger and more consistent associations that could inform interventions and guidelines to improve sleep health in pregnancy.

## Figures and Tables

**Table 1:** Maternal demographic and health-related variables in participants (n = 935).

Variable	Mean ± SD, n (%)
**Age (years old)**	30.0 ± 5.6
**Height (cm)**	158.7 ± 6.4
**Weight (kg)**	69.1 ± 14.7
**Gestational Age**	
**First Trimester**	225 (24.1%)
**Second Trimester**	317 (33.9%)
**Third Trimester**	393 (42.0%)
**Region**	
**Medina City**	249 (26.6%)
**Al-Ahsa Governorate**	214 (22.9%)
**Riyadh City**	172 (18.4%)
**Jeddah City**	102 (10.9%)
**Jazan City**	102 (10.9%)
**Taif City**	96 (10.3%)
**Education**	
**Diploma or Less**	386 (41.3%)
**Undergraduate**	520 (55.6%)
**Postgraduate**	29 (3.1%)
**Occupation**	
**Housewife**	718 (76.8%)
**Public Sector Employee**	68 (7.3%)
**Private Sector Employee**	94 (9.9%)
**Student**	56 (6.0%)
**Currently Smoking**	
**No**	914 (97.8%)
**Yes**	21 (2.2%)
**Chronic Disease**	
**No**	853 (91.2%)
**Yes**	82 (8.8%)
**Have Children**	
**No**	356 (38.1%)
**Yes**	579 (61.9%)
**Total SB (hours/day)**	6.8 ± 3.5
**Sleep Duration (hours/day)**	7.26 ± 2.1
**Sleep Duration Classifications**	
**Optimal-Sleepers (7–9 h/day)**	510 (54.5%)
**Short-Sleepers (<7 h/day)**	308 (32.9%)
**Long-Sleepers (>9 h/day)**	117 (12.5%)

cm, centimeter; kg, kilogram; n, number; SB, sedentary behavior; SD, standard deviation.

**Table 2:** Odds ratios (OR) of poor sleep quality per h/day of SB in pregnant women (n = 935).

Variables	Total SB (h/Day) *	Total SB (h/Weekday) #	Total SB (h/Weekend Day) #	Leisure SB (h/Day) §	Leisure SB (h/Weekday) ∫	Leisure SB (h/Weekend Day) ∫	Occupational SB (h/Day) §	Occupational SB (h/Weekday) ∫	Occupational SB (h/Weekend Day) ∫	Commuting SB (h/Day) §	Commuting SB (h/ Weekday) ∫	Commuting SB (h/Weekend Day) ∫
	All Trimesters (n = 935)											
**OR (95% confidence interval)**	1.031 (0.992, 1.071)	1.005 (0.957, 1.055)	0.963 (0.904, 1.027)	1.020 (0.972, 1.070)	0.963 (0.904, 1.027)	**1.074** **(1.005, 1.148)**	1.092 (0.974, 1.222)	1.095 (0.992, 1.208)	0.902 (0.737, 1.105)	1.040 (0.905, 1.196)	1.030 (0.881, 1.205)	0.997 (0.868, 1.144)
**First Trimester (n = 225)**												
**OR (95% confidence interval)**	1.091 (0.994, 1.196)	0.966 (0.856, 1.090)	**1.142** **(1.017, 1.283)**	1.060 (0.952, 1.188)	0.862 (0.725, 1.024)	1.071 (0.961, 1.194)	1.220 (0.919, 1.622)	1.239 (0.974, 1.575)	1.364 (0.960, 1.939)	1.184 (0.882, 1.590)	0.842 (0.556, 1.275)	1.364 (0.960, 1.939)
**Second Trimester (n = 317)**												
**OR (95% confidence interval)**	1.091 (0.951, 1.082)	0.999 (0.916, 1.091)	1.016 (0.933, 1.106)	0.997 (0.917, 1.083)	0.947 (0.849, 1.056)	1.071 (0.961, 1.194)	1.106 (0.911, 1343)	1.109 (0.929, 1.324)	0.924 (0.730, 1.169)	1.020 (0.804, 1.293)	1.087 (0.830, 1.424)	0.924 (0.730, 1.169)
**Third Trimester (n = 393)**												
**OR (95% confidence interval)**	1.013 (0.954, 1.075)	1.028 (0.957, 1.104)	0.978 (0.901, 1.062)	1.015 (0.941, 1.095)	1.016 (0.923, 1.118)	0.997 (0.897, 1.110)	1.066 (0.891, 1.276)	1.065 (0.919, 1.234)	0.947 (0.687, 1.305)	0.959 (0.765, 1.204)	1.002 (0.794, 1.266)	0.948 (0.765, 1.176)

* adjusted for age, smoking status, moderate-to-vigorous physical activity, having children, education, occupation, and chronic disease status. # adjusted for age, smoking status, moderate-to-vigorous physical activity, having children, education, occupation, chronic disease status, and simultaneously adjusted for total sedentary behavior per weekday and weekend. § adjusted for age, smoking status, moderate-to-vigorous physical activity, having children, education, occupation, chronic disease status, and simultaneously adjusted for the other domain-specific sedentary behavior. ∫ adjusted for age, smoking status, moderate-to-vigorous physical activity, having children, education, occupation, chronic disease status, and simultaneously adjusted for the other patterns and domain-specific sedentary behavior. Bold indicates significant association (*p* < 0.05).

**Table 3:** Odds ratios (OR) of not adhering to sleep duration guidelines per h/day of SB in pregnant women (n = 935).

Variables	Total SB (h/Day) *	Total SB (h/Weekday) #	Total SB (h/Weekend Day) #	Leisure SB (h/Day) §	Leisure SB (h/Weekday) ∫	Leisure SB (h/Weekend Day) ∫	Occupational SB (h/Day) §	Occupational SB (h/Weekday) ∫	Occupational SB (h/Weekend Day) ∫	Commuting SB (h/Day) §	Commuting SB (h/ weekday) ∫	Commuting SB (h/Weekend Day) ∫
	All Trimesters (n = 935)											
**OR (95% confidence interval)**	0.986 (0.950, 1.023)	**1.055** **(1.007, 1.106)**	**0.920** **(0.875, 0.967)**	1.014 (0.968, 1.062)	**1.073** **(1.010, 1.141)**	**0.934** **(0.876, 0.996)**	0.930 (0.829, 1.044)	0.998 (0.905, 1.102)	0.835 (0.676, 1.031)	0.895 (0.782, 1.024)	1.037 (0.891, 1.206)	**0.863** **(0.754, 0.988)**
**First Trimester (n = 225)**												
**OR (95% confidence interval)**	0.945 (0.867, 1.030)	1.027 (0.919, 1.147)	0.912 (0.818, 1.016)	1.026 (0.925, 1.137)	1.100 (0.942, 1.284)	0.922 (0.788, 1.079)	0.788 (0.587, 1.058)	1.239 (0.974, 1.575)	0.757 (0.458, 1.250)	**0.656** **(0.485, 0.886)**	0.842 (0.556, 1.275)	0.876 (0.633, 1.214)
**Second Trimester (n = 317)**												
**OR (95% confidence interval)**	0.990 (0.931, 1.053)	1.025 (0.943, 1.113)	0.962 (0.886, 1.045)	0.980 (0.906, 1.060)	1.020 (0.923, 1.127)	0.961 (0.867, 1.065)	0.952 (0.785, 1.155)	1.014 (0.851, 1.207)	0.861 (0.607, 1.222)	1.076 (0.858, 1.349)	1.100 (0.845, 1.431)	0.900 (0.795, 1.234)
**Third Trimester (n = 393)**												
**OR (95% confidence interval)**	1.004 (0.947, 1.064)	**1.109** **(1.032, 1.191)**	**0.868** **(0.797, 0.945)**	1.026 (0.954, 1.103)	**1.114** **(1.011, 1.228)**	**0.891** **(0.800, 0.994)**	1.000 (0.838, 1.193)	1.050 (0.908, 1.215)	0.832 (0.594, 1.165)	0.900 (0.723, 1.121)	1.141 (0.907, 1.436)	**0.754** **(0.607, 0.938)**

* adjusted for age, smoking status, moderate-to-vigorous physical activity, having children, education, occupation, and chronic disease status. # adjusted for age, smoking status, moderate-to-vigorous physical activity, having children, education, occupation, chronic disease status, and simultaneously adjusted for total sedentary behavior per weekday and weekend. § adjusted for age, smoking status, moderate-to-vigorous physical activity, having children, education, occupation, chronic disease status, and simultaneously adjusted for the other domain-specific sedentary behavior. ∫ adjusted for age, smoking status, moderate-to-vigorous physical activity, having children, education, occupation, chronic disease status, and simultaneously adjusted for the other patterns and domain-specific sedentary behavior. Bold indicates significant association (*p* < 0.05).

**Table 4:** Odds ratios (OR) of being a short-sleeper (n = 308) or long-sleeper (n = 117) compared to being an optimal-duration-sleeper (n = 510) per h/day of SB in pregnant women.

Variables	Total SB (h/Day) *	Total SB (h/Weekday) #	Total SB (h/Weekend Day) #	Leisure SB (h/Day) §	Leisure SB (h/Weekday) ∫	Leisure SB (h/Weekend Day) ∫	Occupational SB (h/Day) §	Occupational SB (h/Weekday) ∫	Occupational SB (h/Weekend Day) ∫	Commuting SB (h/Day) §	Commuting SB (h/ Weekday) ∫	Commuting SB (h/Weekend Day) ∫
	All Trimesters (n = 935)											
**OR of short-sleeper**	**0.957** **(0.917,** **0.998)**	**1.054** **(1.000, 1.110)**	**0.887** **(0.839, 0.939)**	0.972 (0.922, 1.025)	1.043 (0.975, 1.117)	**0.919** **(0.855, 0.987)**	0.995 (0.884, 1.119)	1.043 (0.942, 1.154)	0.865 (0.700, 1.070)	**0.853** **(0.731, 0.996)**	1.082 (0.917, 1.277)	**0.776** **(0.665, 0.906)**
**OR of long-sleeper**	1.055 (1.000, 1.113)	1.057 (0.985, 1.133)	0.995 (0.926, 1.070)	**1.121** **(1.050, 1.196)**	**1.131** **(1.037, 1.234)**	0.988 (0.902, 1.082)	**0.619** **(0.446, ** **0.859)**	0.807 (0.631, 1.033)	0.538 (0.273, 1.061)	0.981 (0.809, 1.190)	0.901 (0.705, 1.152)	1.101 (0.897, 1.350)
**First Trimester (n = 225)**												
**OR of short-sleeper**	**0.871** **(0.788, 0.963)**	1.023 (0.903, 1.159)	**0.832** **(0.733, 0.945)**	0.949 (0.844, 1.067)	1.074 (0.908, 1.271)	0.862 (0.726, 1.023)	0.824 (0.606, 1.121)	0.928 (0.728, 1.183)	0.744 (0.435, 1.273)	**0.562** **(0.387, 0.817)**	0.833 (0.536, 1.294)	0.688 (0.464, 1.021)
**OR of long-sleeper**	1.124 (0.994, 1.272)	1.050 (0.898, 1.228)	1.074 (0.923, 1.251)	**1.232** **(1.066, 1.424**	1.200 (0.961, 1.498)	1.041 (0.840, 1.291)	0.673 (0.361, 1.254)	0.738 (0.416, 1.308)	0.844 (0.349, 2.038)	0.794 (0.542, 1.164)	**0.548** **(0.311, 0.966)**	1.402 (0.892, 2.202)
**Second Trimester (n = 317)**												
**OR of short-sleeper**	0.972 (0.907, 1.041)	1.026 (0.938, 1.123)	0.940 (0.858, 1.029)	0.954 (0.873, 1.043)	1.003 (0.899 ,1.118)	0.949 (0.847, 1.063)	1.038 (0.853, 1.262)	1.058 (0.887, 1.261)	0.942 (0.663, 1.339)	0.998 (0.777, 1.282)	1.109 (0.834, 1.475)	0.906 (0.707, 1.161)
**OR of long-sleeper**	1.040 (0.951, 1.138)	1.030 (0.908, 1.169)	1.010 (0.893, 1.142)	1.058 (0.944, 1.186)	1.050 (0.904, 1.220)	1.013 (0.874, 1.174)	0.617 (0.855, 1.073)	0.853 (0.584, 1.246)	0.270 (0.042, 1.750)	1.269 (0.921, 1.748)	1.218 (0.830, 1.7880	1.068 (0.773, 1.474)
**Third Trimester (n = 393)**												
**OR of short-sleeper**	0.989 (0.928, 1.054)	**1.094** **(1.013,** **1.181)**	**0.869** **(0.793,** **0.952)**	0.992 (0.914, 1.076)	1.060 (0.952, 1.181)	0.915 (0.812, 1.031)	1.048 (0.878, 1.250)	1.076 (0.926, 1.250)	0.867 (0.624, 1.203)	0.923 (0.726, 1.174)	1.205 (0.946, 1.534)	**0.713** **(0.560,** **0.907)**
**OR of long-sleeper**	1.029 (0.941, 1.126)	1.078 (0.986, 1.223)	0.910 (0.799, 1.037)	**1.119** **(1.003, 1.248)**	**1.206** **(1.052,** **1.383)**	0.901 (0.770, 1.054)	0.604 (0.349, 1.045)	0.826 (0.562, 1.215)	0.426 (0.121, 1.495)	0.835 (0.584, 1.193)	0.853 (0.562, 1.296)	0.985 (0.688, 1.411)

* adjusted for age, smoking status, moderate-to-vigorous physical activity, having children, education, occupation, and chronic disease status. # adjusted for age, smoking status, moderate-to-vigorous physical activity, having children, education, occupation, chronic disease status, and simultaneously adjusted for total sedentary behavior per weekday and weekend. § adjusted for age, smoking status, moderate-to-vigorous physical activity, having children, education, occupation, chronic disease status, and simultaneously adjusted for the other domain-specific sedentary behavior. ∫ adjusted for age, smoking status, moderate-to-vigorous physical activity, having children, education, occupation, chronic disease status, and simultaneously adjusted for the other patterns and domain-specific sedentary behavior. Bold indicates significant association (*p* < 0.05).

**Table 5:** Key significant associations between SB and sleep quality and duration in pregnant women (n = 935).

Trimester	Associations	Statistics
All trimesters	Leisure SB (h/weekend day) and poor sleep quality	OR (95% CI): 1.074 (1.005, 1.148)
First trimester	Total SB (h/weekday) and poor sleep quality	OR (95% CI): 1.142 (1.017, 1.283)
All trimesters	Total SB (h/weekday) and not adhering to sleep duration guidelines	OR (95% CI): 1.055 (1.007, 1.106)
All trimesters	Leisure SB (h/weekday) and not adhering to sleep duration guidelines	OR (95% CI): 1.073 (1.010, 1.141)
All trimesters	Total SB (h/weekend day) and not adhering to sleep duration guidelines	OR (95% CI): 0.920 (0.875, 0.967)
All trimesters	Leisure SB (h/weekend day) and not adhering to sleep duration guidelines	OR (95% CI): 0.934 (0.876, 0.996)
All trimesters	Commuting SB (h/weekend day) and not adhering to sleep duration guidelines	OR (95% CI): 0.863 (0.754, 0.988)
First trimester	Commuting SB (h/day) and not adhering to sleep duration guidelines	OR (95% CI): 0.656 (0.485, 0.886)
Third trimester	Total SB (h/weekday) and not adhering to sleep duration guidelines	OR (95% CI): 1.109 (1.032, 1.191)
Third trimester	Leisure SB (h/weekday) and not adhering to sleep duration guidelines	OR (95% CI): 1.114 (1.011, 1.228)
Third trimester	Total SB (h/weekend day) and not adhering to sleep duration guidelines	OR (95% CI): 0.868 (0.797, 0.945)
Third trimester	Leisure SB (h/weekend day) and not adhering to sleep duration guidelines	OR (95% CI): 0.891 (0.800, 0.994)
Third trimester	Commuting SB (h/weekend day) and not adhering to sleep duration guidelines	OR (95% CI): 0.754 (0.607, 0.938)
All trimesters	Total SB (h/day) and being a short-sleeper	OR (95% CI): 0.957 (0.917, 0.998)
All trimesters	Total SB (h/weekend day) and being a short-sleeper	OR (95% CI): 0.887 (0.839, 0.939)
All trimesters	Leisure SB (h/weekend day) and being a short-sleeper	OR (95% CI): 0.919 (0.855, 0.987)
All trimesters	Commuting SB (h/day) and being a short-sleeper	OR (95% CI): 0.853 (0.731, 0.966)
All trimesters	Commuting SB (h/weekend day) and being a short-sleeper	OR (95% CI): 0.776 (0.665, 0.905)
All trimesters	Total SB (h/weekday) and being a short-sleeper	OR (95% CI): 1.054 (1.000, 1.110)
First Trimester	Total SB (h/day) and being a short-sleeper	OR (95% CI): 0.871 (0.788, 0.963)
First Trimester	Total SB (h/weekend day) and being a short-sleeper	OR (95% CI): 0.832 (0.733, 0.945)
First Trimester	Commuting SB (h/day) and being a short-sleeper	OR (95% CI): 0.562 (0.387, 0.817)
Third Trimester	Total SB (h/weekend day) and being a short-sleeper	OR (95% CI): 0.869 (0.793, 0.952)
Third Trimester	Commuting SB (h/weekend day) and being a short-sleeper	OR (95% CI): 0.713 (0.560, 0.907)
Third Trimester	Total SB (h/weekday) and being a short-sleeper	OR (95% CI): 1.094 (1.013, 1.181)
All trimesters	Occupational SB (h/day) and being a long-sleeper	OR (95% CI): 0.619 (0.446, 0.859)
All trimesters	Leisure SB (h/day) and being a long-sleeper	OR (95% CI): 1.121 (1.050, 1.196)
All trimesters	Leisure SB (h/weekday) and being a long-sleeper	OR (95% CI): 1.131 (1.037, 1.234)
First Trimester	Commuting SB (h/weekday) and being a long-sleeper	OR (95% CI): 0.548 (0.311, 0.966)
First Trimester	Leisure SB (h/day) and being a long-sleeper	OR (95% CI): 1.232 (1.066, 1.424)
Third Trimester	Leisure SB (h/day) and being a long-sleeper	OR (95% CI): 1.119 (1.003, 1.248)
Third Trimester	Leisure SB (h/weekday) and being a long-sleeper	OR (95% CI): 1.206 (1.052, 1.383)

## Data Availability

The data are available upon request from the corresponding author.

## References

[1] Lloyd-Jones D.M., Allen N.B., Anderson C.A., Black T., Brewer L.C., Foraker R.E., Grandner M.A., Lavretsky H., Perak A.M., Sharma G. (2022). Life’s essential 8: Updating and enhancing the American Heart Association’s construct of cardiovascular health: A presidential advisory from the American Heart Association. Circulation.

[2] Okoli A., Hanlon E.C., Brady M.J. (2021). The relationship between sleep, obesity, and metabolic health in adolescents: A review. Curr. Opin. Endocr. Metab. Res..

[3] Ogilvie R.P., Patel S.R. (2018). The epidemiology of sleep and diabetes. Curr. Diabetes Rep..

[4] Hsieh C.G., Martin J.L. (2019). Short sleep, insomnia, and cardiovascular disease. Curr. Sleep Med. Rep..

[5] Lu Q., Zhang X., Wang Y., Li J., Xu Y., Song X., Su S., Zhu X., Vitiello M.V., Shi J. (2021). Sleep disturbances during pregnancy and adverse maternal and fetal outcomes: A systematic review and meta-analysis. Sleep Med. Rev..

[6] Wang R., Xu M., Yang W., Xie G., Yang L., Shang L., Zhang B., Guo L., Yue J., Zeng L. (2022). Maternal sleep during pregnancy and adverse pregnancy outcomes: A systematic review and meta-analysis. J. Diabetes Investig..

[7] Gonzalez-Mesa E., Cuenca-Marin C., Suarez-Arana M., Tripiana-Serrano B., Ibrahim-Diez N., Gonzalez-Cazorla A., Blasco-Alonso M. (2019). Poor sleep quality is associated with perinatal depression. A systematic review of last decade scientific literature and meta-analysis. J. Perinat. Med..

[8] Lawson A., Murphy K.E., Sloan E., Uleryk E., Dalfen A. (2015). The relationship between sleep and postpartum mental disorders: A systematic review. J. Affect. Disord..

[9] Sedov I.D., Cameron E.E., Madigan S., Tomfohr-Madsen L.M. (2018). Sleep quality during pregnancy: A meta-analysis. Sleep Med. Rev..

[10] Xu X., Liu D., Zhang Z., Sharma M., Zhao Y. (2017). Sleep duration and quality in pregnant women: A cross-sectional survey in China. Int. J. Environ. Res. Public Health.

[11] Feinstein L., McWhorter K.L., Gaston S.A., Troxel W.M., Sharkey K.M., Jackson C.L. (2020). Racial/ethnic disparities in sleep duration and sleep disturbances among pregnant and non-pregnant women in the United States. J. Sleep Res..

[12] Alghamdi S.A., Alsalman A., Sowadi O.K., Khojah N., Saad H., Gibbs B.B., Alshuwaier G.O., Alansare A.B. (2024). Compliance with 24 h Movement Behavior Guidelines for Pregnant Women in Saudi Arabia: The Role of Trimester and Maternal Characteristics. Healthcare.

[13] Bacaro V., Benz F., Pappaccogli A., De Bartolo P., Johann A.F., Palagini L., Lombardo C., Feige B., Riemann D., Baglioni C. (2020). Interventions for sleep problems during pregnancy: A systematic review. Sleep Med. Rev..

[14] Paulino D.S.M., Borrelli C.B., Faria-Schützer D.B., Brito L.G.O., Surita F.G. (2022). Non-pharmacological interventions for improving sleep quality during pregnancy: A systematic review and meta-analysis. Rev. Bras. De Ginecol. E Obs..

[15] Okun M.L., Ebert R., Saini B. (2015). A review of sleep-promoting medications used in pregnancy. Am. J. Obstet. Gynecol..

[16] Coll C.V., Domingues M.R., Gonçalves H., Bertoldi A.D. (2017). Perceived barriers to leisure-time physical activity during pregnancy: A literature review of quantitative and qualitative evidence. J. Sci. Med. Sport.

[17] Tremblay M.S., Aubert S., Barnes J.D., Saunders T.J., Carson V., Latimer-Cheung A.E., Chastin S.F., Altenburg T.M., Chinapaw M.J. (2017). Sedentary behavior research network (SBRN)–terminology consensus project process and outcome. Int. J. Behav. Nutr. Phys. Act..

[18] Fazzi C., Saunders D.H., Linton K., Norman J.E., Reynolds R.M. (2017). Sedentary behaviours during pregnancy: A systematic review. Int. J. Behav. Nutr. Phys. Act..

[19] Hawkins M., Kim Y., Gabriel K.P., Rockette-Wagner B.J., Chasan-Taber L. (2017). Sedentary behavior patterns in non-pregnant and pregnant women. Prev. Med. Rep..

[20] Katzmarzyk P.T., Powell K.E., Jakicic J.M., Troiano R.P., Piercy K., Tennant B., Committee P.A.G.A. (2019). Sedentary behavior and health: Update from the 2018 physical activity guidelines advisory committee. Med. Sci. Sports Exerc..

[21] Osumi A., Kanejima Y., Ishihara K., Ikezawa N., Yoshihara R., Kitamura M., Izawa K.P. (2024). Effects of Sedentary Behavior on the Complications Experienced by Pregnant Women: A Systematic Review. Reprod. Sci..

[22] Lane A., Catov J., Jones M.A., Gibbs B.B. (2022). Patterns in Prenatal Physical Activity and Sedentary Behavior: Associations With Blood Pressure and Placental Features in the MoMHealth Cohort. J. Phys. Act. Health.

[23] Loprinzi P.D., Fitzgerald E.M., Woekel E., Cardinal B.J. (2013). Association of physical activity and sedentary behavior with biological markers among US pregnant women. J. Women’s Health.

[24] Reid E.W., McNeill J.A., Alderdice F.A., Tully M.A., Holmes V.A. (2014). Physical activity, sedentary behaviour and fetal macrosomia in uncomplicated pregnancies: A prospective cohort study. Midwifery.

[25] Gibbs B.B., Jones M.A., Jakicic J.M., Jeyabalan A., Whitaker K.M., Catov J.M. (2021). Objectively measured sedentary behavior and physical activity across 3 trimesters of pregnancy: The monitoring movement and health study. J. Phys. Act. Health.

[26] Bull F.C., Al-Ansari S.S., Biddle S., Borodulin K., Buman M.P., Cardon G., Carty C., Chaput J.-P., Chastin S., Chou R. (2020). World Health Organization 2020 guidelines on physical activity and sedentary behaviour. Br. J. Sports Med..

[27] Evenson K.R., Brown W.J., Brinson A.K., Budzynski-Seymour E., Hayman M. (2023). A review of public health guidelines for postpartum physical activity and sedentary behavior from around the world. J. Sport Health Sci..

[28] Gubelmann C., Heinzer R., Haba-Rubio J., Vollenweider P., Marques-Vidal P. (2018). Physical activity is associated with higher sleep efficiency in the general population: The CoLaus study. Sleep.

[29] Koohsari M.J., Yasunaga A., McCormack G.R., Shibata A., Ishii K., Liao Y., Nagai Y., Oka K. (2023). Sedentary behaviour and sleep quality. Sci. Rep..

[30] Vallance J.K., Buman M.P., Stevinson C., Lynch B.M. (2015). Associations of overall sedentary time and screen time with sleep outcomes. Am. J. Health Behav..

[31] Suleiman K.H., Yates B.C., Berger A.M., Pozehl B., Meza J. (2010). Translating the Pittsburgh sleep quality index into Arabic. West. J. Nurs. Res..

[32] Zhong Q.-Y., Gelaye B., Sánchez S.E., Williams M.A. (2015). Psychometric properties of the Pittsburgh Sleep Quality Index (PSQI) in a cohort of Peruvian pregnant women. J. Clin. Sleep Med..

[33] Buysse D.J., Reynolds III C.F., Monk T.H., Berman S.R., Kupfer D.J. (1989). The Pittsburgh Sleep Quality Index: A new instrument for psychiatric practice and research. Psychiatry Res..

[34] Watson N.F., Badr M.S., Belenky G., Bliwise D.L., Buxton O.M., Buysse D., Dinges D.F., Gangwisch J., Grandner M.A., Consensus Conference Panel (2015). Joint consensus statement of the American Academy of Sleep Medicine and Sleep Research Society on the recommended amount of sleep for a healthy adult: Methodology and discussion. J. Clin. Sleep Med..

[35] Alaqil A.I., Gupta N., Alothman S.A., Al-Hazzaa H.M., Stamatakis E., del Pozo Cruz B. (2023). Arabic translation and cultural adaptation of sedentary behavior, dietary habits, and preclinical mobility limitation questionnaires: A cognitive interview study. PLoS ONE.

[36] Oviedo-Caro M.Á., Bueno-Antequera J., Munguía-Izquierdo D. (2018). Measuring sedentary behavior during pregnancy: Comparison between self-reported and objective measures. Matern. Child Health J..

[37] Rosenberg D.E., Norman G.J., Wagner N., Patrick K., Calfas K.J., Sallis J.F. (2010). Reliability and validity of the Sedentary Behavior Questionnaire (SBQ) for adults. J. Phys. Act. Health.

[38] Mielke G.I., Crochemore M da Silva I., Gomersall S.R., Owen N., Hallal P.C. (2020). Reliability of a multi-domain sedentary behaviour questionnaire and comparability to an overall sitting time estimate. J. Sports Sci..

[39] Craig C.L., Marshall A.L., Sjöström M., Bauman A.E., Booth M.L., Ainsworth B.E., Pratt M., Ekelund U., Yngve A., Sallis J.F. (2003). International physical activity questionnaire: 12-country reliability and validity. Med. Sci. Sports Exerc..

[40] Sanda B., Vistad I., Haakstad L.A.H., Berntsen S., Sagedal L.R., Lohne-Seiler H., Torstveit M.K. (2017). Reliability and concurrent validity of the International Physical Activity Questionnaire short form among pregnant women. BMC Sports Sci. Med. Rehabil..

[41] Tantrakul V., Ingsathit A., Liamsombut S., Rattanasiri S., Kittivoravitkul P., Imsom-Somboon N., Lertpongpiroon S., Jantarasaengaram S., Somchit W., Suwansathit W. (2023). Treatment of obstructive sleep apnea in high risk pregnancy: A multicenter randomized controlled trial. Respir. Res..

[42] Okun M.L., Tolge M., Hall M. (2014). Low socioeconomic status negatively affects sleep in pregnant women. J. Obstet. Gynecol. Neonatal Nurs..

[43] Elbarazi I., Alam Z., Ali N., Loney T., Al-Rifai R.H., Al-Maskari F., Ahmed L.A. (2024). Health literacy among pregnant women in the United Arab Emirates: The Mutaba’ah study. Women’s Health.

[44] Golzar J., Noor S., Tajik O. (2022). Convenience sampling. Int. J. Educ. Lang. Stud..

[45] Healy G.N., Clark B.K., Winkler E.A., Gardiner P.A., Brown W.J., Matthews C.E. (2011). Measurement of adults’ sedentary time in population-based studies. Am. J. Prev. Med..

[46] Wilson D.L., Fung A., Walker S.P., Barnes M. (2013). Subjective reports versus objective measurement of sleep latency and sleep duration in pregnancy. Behav. Sleep Med..

[47] Xu X., Liu D., Rao Y., Zeng H., Zhang F., Wang L., Xie Y., Sharma M., Zhao Y. (2018). Prolonged Screen Viewing Times and Sociodemographic Factors among pregnant women: A cross-sectional survey in China. Int. J. Environ. Res. Public Health.

[48] Wagnild J.M., Pollard T.M. (2021). How is television time linked to cardiometabolic health in adults? A critical systematic review of the evidence for an effect of watching television on eating, movement, affect and sleep. BMJ Open.

[49] Custers K., Van den Bulck J. (2012). Television viewing, internet use, and self-reported bedtime and rise time in adults: Implications for sleep hygiene recommendations from an exploratory cross-sectional study. Behav. Sleep Med..

[50] Bakker E.A., Hopman M.T., Lee D.-c., Verbeek A.L., Thijssen D.H., Eijsvogels T.M. (2020). Correlates of Total and domain-specific Sedentary behavior: A cross-sectional study in Dutch adults. BMC Public Health.

[51] Drenowatz C., DeMello M.M., Shook R.P., Hand G.A., Burgess S., Blair S.N. (2016). The association between sedentary behaviors during weekdays and weekend with change in body composition in young adults. AIMS Public Health.

[52] Alansare A.B., Paley J.L., Quinn T.D., Gibbs B.B. (2023). Paradoxical Associations of Occupational and Nonoccupational Sedentary Behavior with Cardiovascular Disease Risk Measures in Desk Workers. J. Occup. Environ. Med..

[53] Kim Y., Wilkens L.R., Park S.-Y., Goodman M.T., Monroe K.R., Kolonel L.N. (2013). Association between various sedentary behaviours and all-cause, cardiovascular disease and cancer mortality: The Multiethnic Cohort Study. Int. J. Epidemiol..

